# Development of Gelatin Methacryloyl/Sodium Alginate Interpenetrating Polymer Network Hydrogels for Bone Regeneration by Activating the Wnt/β-Catenin Signaling Pathway via Lithium Release

**DOI:** 10.3390/ijms241713613

**Published:** 2023-09-02

**Authors:** Chen Ma, Yu-Kyoung Kim, Min-Ho Lee, Yong-Seok Jang

**Affiliations:** Department of Dental Biomaterials, Institute of Biodegradable Materials, School of Dentistry, Jeonbuk National University, Jeon-Ju 54896, Republic of Korea; 1995machen@gmail.com (C.M.); yk0830@naver.com (Y.-K.K.); mh@jbnu.ac.kr (M.-H.L.)

**Keywords:** interpenetrating polymer network hydrogel, alginate, gelatin methacryloyl, lithium, mechanical properties, biocompatibility

## Abstract

Hydrogels have gained significant attention as biomaterials due to their remarkable properties resembling those of the extracellular matrix (ECM). In the present investigation, we successfully synthesized interpenetrating polymer network (IPN) hydrogels using gelatin methacryloyl (GelMA) and sodium alginate (SA), incorporating various concentrations of lithium chloride (LiCl; 0, 5, and 10 mM), aiming to develop a hydrogel scaffold for bone regeneration. Notably, the compressive modulus of the IPN hydrogels remained largely unaffected upon the inclusion of LiCl. However, the hydrogel with the high concentration of LiCl exhibited reduced fragmentation after compression testing. Intriguingly, we observed a significant improvement in cellular biocompatibility, primarily attributed to activation of the Wnt/β-catenin signaling pathway induced by LiCl. Subsequently, we evaluated the efficacy of the newly developed IPN-Li hydrogels in a rat cranial defect model and found that they substantially enhanced bone regeneration. Nevertheless, it is important to note that the introduction of high concentrations of LiCl did not significantly promote osteogenesis. This outcome can be attributed to the excessive release of Li^+^ ions into the extracellular matrix, hindering the desired effect. Overall, the IPN-Li hydrogel developed in this study holds great promise as a biodegradable material for bone regeneration applications.

## 1. Introduction

Hydrogels have emerged as valuable materials for medical applications, including drug release, medical dressings, gingival tissue regeneration, and bone repair, owing to their remarkable mechanical and bioactive properties resembling those of the extracellular matrix (ECM). Significant research efforts have been devoted to bone regeneration, resulting in notable studies such as the development of an injectable hydrogel for long bone defects [1], a porous hydrogel for cartilage defects [2], and the investigation of a hydrogel for skeletal muscle [3].

GelMA, a semi-natural synthetic material derived from gelatin, exhibits high biocompatibility, biodegradability, and low antigenicity. Notably, GelMA contains the arginine-glycine-aspartate (RGD) sequence, which significantly enhances cell adhesion [4]. However, GelMA hydrogels are limited in their application as medical materials due to their low viscosity, resulting in fragility and inferior mechanical properties compared to other hydrogels. Due to MA grafting, GelMA has plasticity and controlled-release capabilities under light curing; therefore, various photoinitiators have been utilized for GelMA crosslinking, such as Irgacure 2959 (UV, 365 nm) [5], LAP (visible light, 405 nm) [6], and Eosin Y (visible light, 490–510 nm) [7]. In this study, we employed the commonly used crosslinker, Irgacure 2959, due to its moderate water solubility, biocompatibility, and low immunogenicity [8]. To enhance the mechanical properties of GelMA hydrogels for long-term bone regeneration applications, we introduced alginate, a polymer with a low degradation rate, to synthesize an interpenetrating polymer network (IPN) hydrogel. Alginate, derived from brown seaweed, is widely utilized in hydrogel biomaterials due to its excellent characteristics, including biocompatibility, non-toxicity, and gentle gelation. Alginate is composed of two important components, β-D-mannuronic acid (M) and β-L-guluronic acid (G), linked by 1–4 bonds. When exposed to polyvalent metal cations such as Ca^2+^, Sr^2+^, Fe^3+^, and Al^3+^, the G-monomer blocks in the alginate polymer chains form ionic bridges, resulting in the formation of alginate salt hydrogels [9,10,11,12]. Although alginate hydrogels exhibit limited degradability, the presence of divalent cations facilitates the gradual replacement of monovalent cations (Na^+^) in the surrounding medium, thereby accelerating the degradation of sodium alginate. Hence, alginate holds promise as a material for drug delivery systems [13]. However, the absence of the RGD sequence in alginate hinders cell attachment, despite its hydrophilic nature facilitating the adsorption of small amounts of proteins. Biocompatible samples with excellent mechanical properties were synthesized by combining GelMA and alginate in this study.

The loading of small molecules into biomaterials has been extensively explored, as it enables targeted and sustained release of these molecules during tissue repair [14]. However, the field of small molecule tissue-engineered scaffolds for bone regeneration is still in its early stages, with limited research on such biomaterials. Several ions, including boron (B^3+^), calcium (Ca^2+^), copper (II) (Cu^2+^), lithium (Li^+^), magnesium (Mg^2+^), silver (Ag^+^), strontium (Sr^2+^), and zinc (Zn^2+^), have demonstrated the ability to induce osteoblast differentiation through growth factor signaling pathways or by stimulating other processes that support bone tissue regeneration [15,16,17,18,19,20,21,22]. The use of small molecule ions to induce bone tissue repair offers numerous advantages over protein growth factors, including cost effectiveness, simplicity, greater stability, and efficacy at lower concentrations [23]. The Wnt/β-catenin signaling pathway is important for bone formation, and especially in osteocytes, this pathway is essential for viability, protection against apoptotic factors, and communication [24]. Modulating the Wnt signaling pathway has emerged as a promising approach for treating diverse diseases. Inhibiting the Wnt signaling pathway shows potential in cancer treatment by stimulating anti-tumor immunity and impeding cancer progression [25]. On the other hand, positive regulation of the Wnt pathway plays a crucial role in addressing specific conditions like osteoporosis; it offers a promising avenue for mitigating the effects of bone loss by regulating osteoblast differentiation and survival [26]. Furthermore, in the context of osteoarthritis, the Wnt signaling pathway contributes to the regulation of arthritis pathogenesis in tissues containing cartilage, offering potential therapeutic targets for managing osteoarthritis [27]. Lithium ions were applied in this study, as lithium particles inhibit the activity of GSK-3β, which is considered to be a key regulator in the Wnt/β -catenin pathway. Stable β-catenin then aggregates in the cytoplasm and translocates to the nucleus, thereby upregulating osteoblast proliferation and increasing bone formation [18].

In this study, we focused on the development of a hydrogel scaffold loaded with small molecules for long-term bone regeneration. The GelMA alginate IPN hydrogel loaded with Li^+^ ions synthesized in this study possessed good mechanical properties, degradation time, and in vitro and in vivo biocompatibility.

## 2. Results

**Figure 1:**
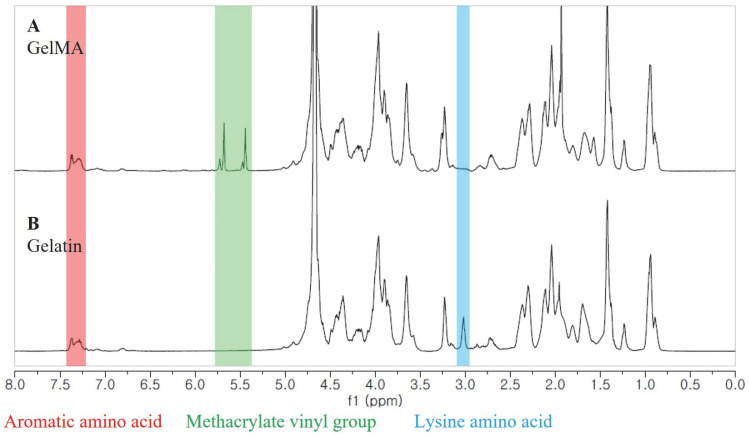
^1^H NMR spectra of GelMA (**A**) and unmodified gelatin (**B**).

We synthesized GelMA using the convenient and efficient one-pot synthesis method reported in a previous study [28]. To investigate the substitution degree of the GelMA samples, we conducted ^1^H NMR analysis. The ^1^H NMR spectra (Figure 1) revealed distinct signals in GelMA compared to unmodified gelatin. Specifically, the new signals at δ = 5.4 and 5.7 ppm in GelMA corresponded to the protons of the methacrylate vinyl group from MA, while the reduced intensity of the peak at δ = 2.9 ppm corresponded to the protons of the methylene group from lysine. Additionally, a peak with constant intensity at δ = 7.3 ppm was observed, representing the aromatic amino acid present in both gelatin and GelMA. To calculate the degree of substitution, we normalized the gelatin and GelMA samples based on the peak intensity of the aromatic amino acid. The degree of substitution for the GelMA sample used in this study was determined to be 91%.

**Figure 2:**
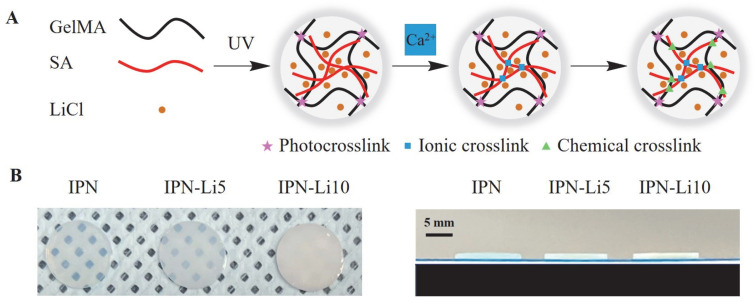
(**A**) Schematic of IPN hydrogels with LiCl synthesized using three crosslinking steps, (**B**) digital photos of IPN hydrogels with different concentrations of LiCl.

Then, we synthesized the IPN-Li hydrogels using a three-step crosslinking method (Figure 2), while a control group was prepared without the inclusion of Li^+^ ions. The resulting IPN-Li hydrogels exhibited uniform texture and regular shape, while the pure IPN hydrogel displayed a high level of transparency. However, as shown in Figure 2B, the transparency of the hydrogel samples significantly decreased with increasing concentration of Li^+^ ions.

**Figure 3:**
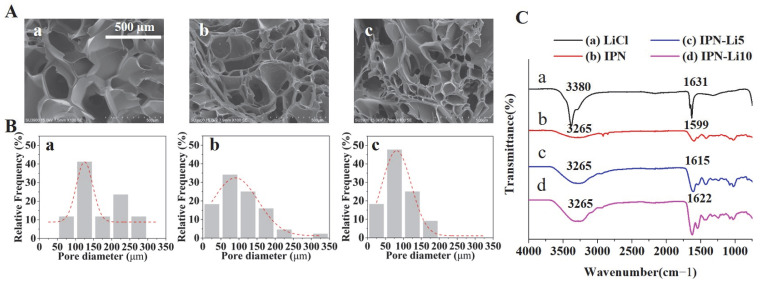
(**A**) SEM images of IPN hydrogels with different concentrations of LiCl, (**B**) pore size distribution analysis in SEM images with different IPN hydrogels using Image J software, (**C**) FTIR spectra of LiCl, IPN, IPN-Li5, and IPN-Li10 hydrogels.

SEM images (Figure 3A) revealed a porous structure in all samples. The pore size was found to be significantly influenced by the concentration of LiCl, with higher LiCl concentrations resulting in smaller pores. Specifically, the pore sizes of the IPN, IPN-5, and IPN-10 hydrogels were measured as 166.7 ± 68.9, 109.6 ± 61.7, and 85.6 ± 42.4 µm, respectively. Furthermore, FTIR analysis (Figure 3C) indicated distinct peaks at 3265, 1599, 1615, and 1622 cm^−1^, corresponding to O-H groups, and the intensities of these peaks increased with higher concentrations of Li+ ions. Additionally, the vibrational band located between 1599 and 1622 cm^−1^, representative of hydroxyl groups, gradually shifted to higher wavenumbers as the concentration of Li^+^ ions increased.

**Figure 4:**
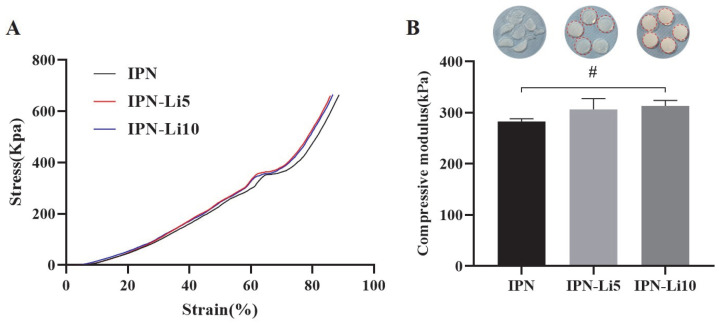
(**A**) Stress–strain curves of IPN hydrogels with different concentrations LiCl, (**B**) hydrogels after compression testing (the red outline indicates the sample is intact) and the compressive moduli of different IPN hydrogels calculated using stress–strain curves (# *p*> 0.05).

To investigate the impact of doping with varying concentrations of Li^+^ ions on the mechanical strength of the IPN hydrogels, we conducted compressive modulus testing on different IPN hydrogel samples. Each group of IPN hydrogels consisted of five samples. Analysis of the stress–strain curves (Figure 4A) revealed that, at approximately 65% strain, the curves abruptly flattened, indicating structural damage to the hydrogels. The stress–strain curves of all IPN hydrogels exhibited a similar trend, and no significant statistical difference was observed in the compressive modulus among the three groups of IPN hydrogels upon calculation (Figure 4B). The compressive moduli were determined as 282.9 ± 5.1 kPa for the IPN hydrogel, 306.5 ± 21.1 kPa for the IPN-Li5 hydrogel, and 313 ± 10.8 kPa for the IPN-Li10 hydrogel. However, it is important to note that while all samples in the IPN group sustained damage, only one sample was damaged in the IPN-Li5 group, and none of the samples were damaged in the IPN-Li10 group.

**Figure 5:**
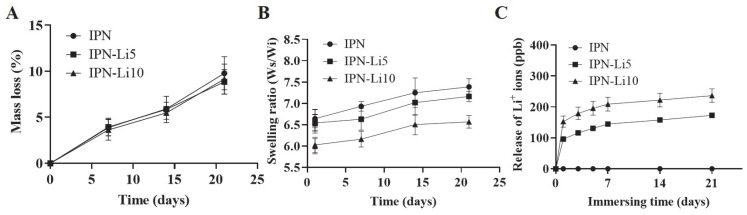
(**A**,**B**) The mass loss and swelling ratio of IPN, IPN-Li5, and IPN-Li10 hydrogels, (**C**) release of Li^+^ ions from different IPN hydrogels measured by ICP-MS.

To evaluate the impact of different concentrations of Li^+^ ions on the stability of the IPN hydrogels, we conducted a 21-day immersion experiment to analyze the degradation rate and swelling ratio of the hydrogels. As depicted in Figure 5A, the addition of varying concentrations of Li^+^ ions did not significantly affect the degradation rate of the IPN hydrogels. The mass loss after 21 days for the IPN, IPN-Li5, and IPN-Li10 hydrogels was measured as 9.8 ± 1.8%, 8.9 ± 1.3%, and 9.1 ± 1.6%, respectively. However, the swelling rate of the IPN hydrogels exhibited a gradual decrease with increasing concentrations of Li^+^ ions. The ratios of wet weight to initial dry weight after 21 days were determined as 7.4 ± 0.2 for IPN, 7.2 ± 0.1 for IPN-Li5, and 6.6 ± 0.1 for IPN-Li10 (Figure 5B). In the ICP-MS experiments (Figure 5C), it was observed that the amount of Li^+^ ions released was proportional to the loading concentration. After 21 days, IPN-Li5 released 172.8 ± 6.1 ppb of Li^+^ ions, while IPN-Li10 released 236.5 ± 21.7 ppb of Li^+^ ions. All Li^+^ ion-doped IPN hydrogels exhibited a similar release trend, characterized by an initial burst release on the first day, followed by a gradual and sustained release. These findings indicated that the IPN hydrogels have the potential to sustain a slow release of Li+ ions for a duration of 21 days.

**Figure 6:**
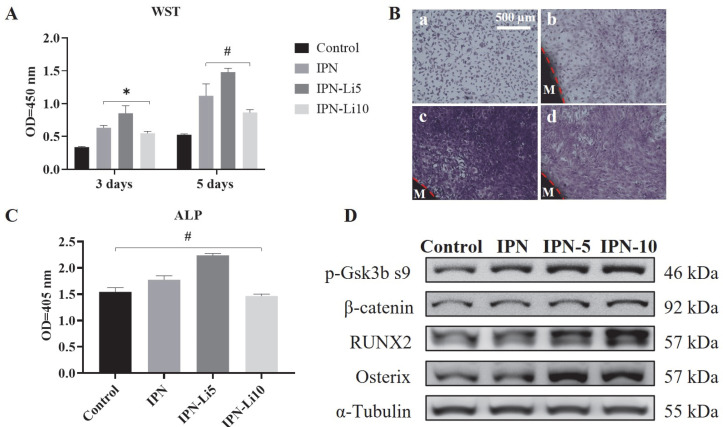
(A) The proliferation of MC3T3-E1 cells co-cultured with different IPN hydrogels after 3 and 5 days through WST assay, (B) crystal violet staining of MC3T3-E1 in control (a), IPN (b), IPN-Li5 (c), and IPN-Li10 (d) groups after 5 days (M represents the hydrogel material), (C) the proliferation of MC3T3-E1 cells co-cultured with different IPN hydrogels after 14 days determined by ALP assay, (D) Western blot analysis of p-GSK-3β s9, β-catenin, RUNX2, and osterix (* *p* < 0.05, # *p* > 0.05).

Regarding cell proliferation, all IPN hydrogel co-cultures exhibited significantly higher values compared to the control group after three days. Particularly noteworthy was the IPN-Li5 group, which demonstrated the highest cell proliferation. However, the IPN hydrogel doped with 10 mM LiCl resulted in relatively lower cell proliferation, albeit still higher than that of the control group. Additionally, the trend in cell proliferation between the 3-day and 5-day cultures remained consistent, with the exception that no statistical difference was observed between the IPN and IPN-10 groups (Figure 6A). To complement the WST assay results, the number and morphology of cells were assessed using crystal violet stain (Figure 6B). The findings aligned with the WST assay results, confirming that the IPN-Li5 group exhibited the most pronounced enhancement in cell proliferation. Subsequently, cell differentiation was evaluated using an ALP kit (Figure 6C). After a 14-day culture period, both the IPN and IPN-Li5 groups displayed significantly higher ALP values compared to the control group, with the IPN-Li5 group exhibiting the greatest enhancement. However, the IPN-10 group did not exhibit a statistically significant difference from the control group. To further explore the impact of different concentrations of LiCl in the IPN hydrogels on cell differentiation, western blot analysis was conducted. The results indicated a positive correlation between Li+ ion concentration and the expression of p-Gsk3β s9, β-catenin, and RUNX2. Notably, the IPN-Li5 group displayed the highest expression of osterix among all groups (Figure 6D).

**Figure 7:**
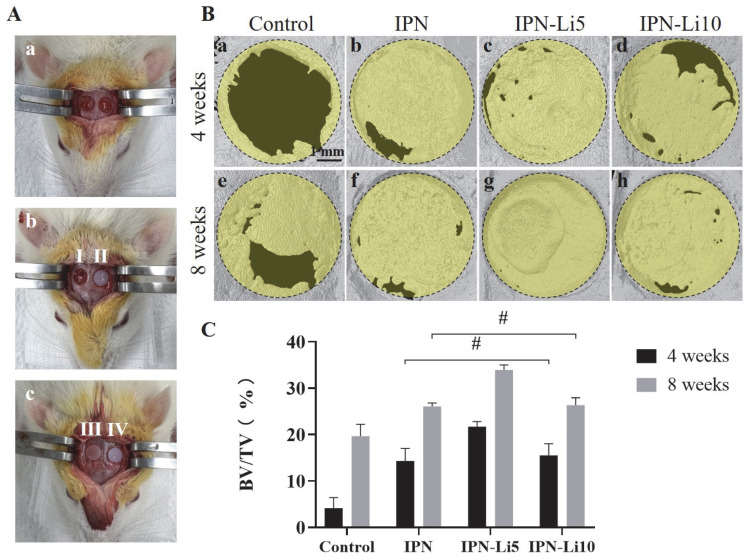
The calvarial bone regeneration after implantation with the hydrogel scaffolds for 4 and 8 weeks in vivo. (**A**) Surgical photos of rat calvarial bone defect and hydrogel implantation (a, calvarial bone defect model; I, II, III, IV in b and c are control without hydrogel and implantation with IPN, IPN-Li5, and IPN-Li10 hydrogels, respectively), (**B**) micro-CT reconstructed images of calvarial defects after implantation for 4 and 8 weeks, (**C**) quantitative analysis of bone tissue volume/total tissue volume (BV/TV) determined by CT Analyzer software (# *p* > 0.05).

Figure 7B presents the results of 3D reconstruction using a rat calvarial bone defect model. After 4 weeks of implantation, only a limited amount of new bone formation was observed at the edge of the defect in the control group (Figure 7B, a). In contrast, the groups implanted with hydrogels exhibited relatively higher levels of new bone formation, particularly the IPN-Li5 group. As the implantation period extended to 8 weeks, the amount of newly formed bone further increased. Notably, the IPN-Li5 group displayed a distinct new bone island, demonstrating excellent osseointegration ability (Figure 7B, g). The quantitative analysis of bone volume fraction (BV/TV) in Figure 7C confirmed the significantly improved bone regeneration following implantation with IPN hydrogels, particularly the IPN-Li5 group. However, there was no statistical difference observed between the IPN and IPN-Li10 groups.

**Figure 8:**
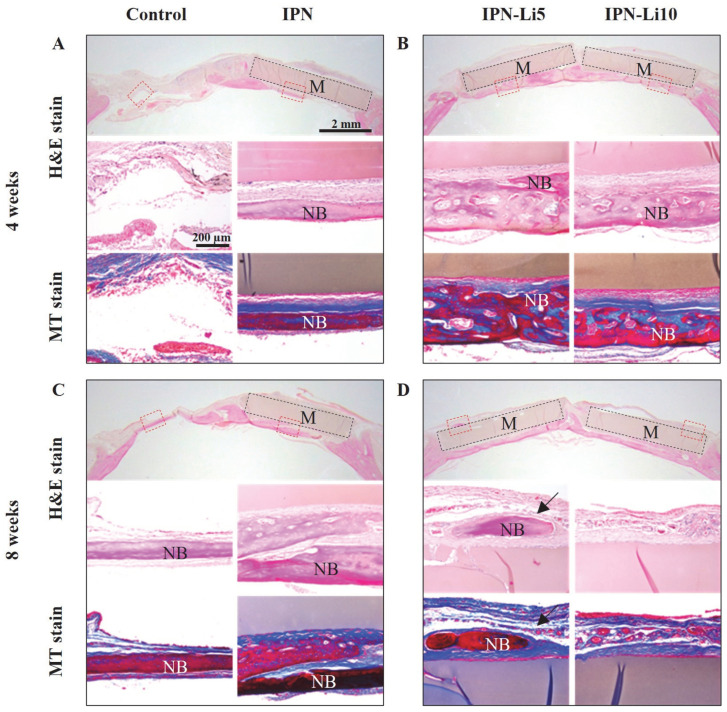
Histological evaluation of calvarial bone regeneration with different IPN hydrogels for 4 (**A**,**B**) and 8 (**C**,**D**) weeks via H&E (**a**–**c**) and MT (**d**,**e**) staining. The blue, dark pink, and light pink colors in the H&E-stained tissue indicate nuclei, bone, and connective tissue, respectively; the blue and red colors in the MT-stained tissue indicate new early bone and mature bone tissue, respectively. The subfigures (**b**–**e**) are enlarged images of the areas in red boxes in the H&E-stained images (**a**); black boxes and M indicate hydrogel materials, NB represents new bone, and the black arrow indicates the bone island.

In the H&E- and MT-stained images (Figure 8), after the fourth week of implantation, the control group exhibited a predominant presence of connective tissue, while the IPN group displayed a small amount of new bone formation below the hydrogel. Notably, the IPN-Li5 group exhibited the thickest and most mature bone tissue, which is visually represented by the red color in the MT-stained images. As the eighth week of implantation was reached, a significant increase in new bone formation was observed in all groups compared to the fourth week. Furthermore, in the IPN-Li5 group, a distinct new bone island (indicated by the black arrow) formed above the implanted hydrogel.

## 3. Discussion

The mechanical strength of GelMA hydrogels is influenced by the degree of substitution, as highlighted in previous studies [29]. GelMA with a higher degree of substitution exhibits a relatively higher compressive modulus due to a decrease in the number of free amino functional groups, resulting in a higher degree of crosslinking. This characteristic aligns with the objective of this study, which aimed to synthesize hydrogels for bone regeneration. Therefore, we prepared GelMA samples with a high degree of substitution (91%) using the one-pot synthesis method with carbonate-bicarbonate buffer. To further enhance the scaffold’s performance, we synthesized interpenetrating network (IPN) hydrogels by combining GelMA and alginate, leveraging their respective advantages. Alginate is well known for its composition of β-D-mannuronic acid (M) and β-L-guluronic acid (G), wherein G-blocks can be crosslinked by divalent cations. Thus, in this study, CaCl_2_ was employed as a crosslinker for alginate, providing Ca^2+^ ions. The triple crosslinking mechanism involved covalent bonding crosslinked by GelMA and the photoinitiator under UV irradiation, crosslinking of G-blocks in alginate by Ca^2+^, and chemical bonding between alginate and GelMA. This synthesis approach resulted in hydrogels with excellent physical properties.

Lithium had an effect on the mechanical properties of the IPN hydrogels in this study. Increasing the concentration of Li^+^ ions led to a slight increase in the compressive modulus of the IPN hydrogels and a significant decrease in the swelling ratio. Similar observations have been reported in previous studies [30]. The reason behind this phenomenon lies in lithium’s ability to replace sodium in the sodium alginate of the IPN hydrogel system through a double-displacement reaction involving both G-blocks and M-blocks [31]. This substitution increases crosslinking, resulting in a smaller pore size structure. Analysis of the FTIR spectra provided insight into the molecular level effects of different concentrations of Li^+^ ions on the IPN hydrogels. The band at 3265 cm^−1^ can be attributed to the O-H stretching of the hydroxyl group, and its intensity increased with increasing Li^+^ concentration, indicating that more O-H groups were produced in the IPN hydrogels. Furthermore, the spectral band between 1599 and 1622 cm^−1^, representing O-H groups, which can be interpreted as the O–H bending vibration of H_2_O, shifted to higher wavenumbers accompanied by an increase in intensity with increasing LiCl loading [32]. Therefore, the more lithium incorporated in the IPN hydrogel, the less sample breakage after compression testing.

**Figure 9:**
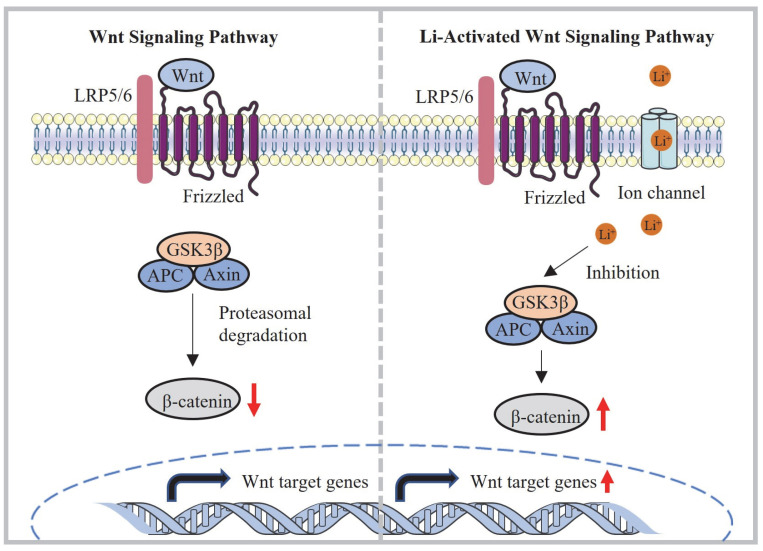
Mechanism of activation of the Wnt/β-catenin signaling pathway via lithium released from the IPN-Li hydrogel.

Cytocompatibility is a crucial factor for evaluating biomaterials, and it plays a significant role in their application. In this study, the cytocompatibility of the IPN hydrogels was assessed to determine their suitability for supporting osteoblast proliferation and differentiation. It has been reported in previous studies [30,33] that appropriate concentrations of Li^+^ ions can promote osteoblast proliferation and differentiation by acting as agonists of the Wnt/β-catenin pathway. Li^+^ ions inhibit GSK-3β, which is a negative regulatory factor in the signaling pathway (Figure 9). Our experimental data confirmed the positive effect of Li^+^ ions on osteoblast behavior. The IPN-Li5 group demonstrated the highest cell proliferation rate at both three and five days of co-culture. This finding suggested that the presence of an optimal concentration of Li^+^ ions promoted osteoblast proliferation. However, it is worth noting that excessive Li^+^ release from the IPN hydrogel had a negative effect on cell proliferation, as observed in the IPN-Li10 group.

Regarding cell differentiation, the IPN-Li5 group exhibited the highest total ALP values after 14 days of co-culture with cells and samples. This outcome indicated that the optimal concentration of Li^+^ ions facilitated osteoblast differentiation. To gain a better understanding of the underlying mechanism, western blot data were analyzed to assess the effect of different concentrations of Li^+^ ions on cell differentiation through the Wnt/β-catenin pathway. GSK3β phosphorylates β-catenin, leading to its inhibition. By adding more Li^+^ ions, GSK3β phosphorylation was increased, resulting in the accumulation of more stable β-catenin. This accumulation of β-catenin promoted the expression of bone-related proteins such as RUNX2 and osterix, further supporting osteoblast differentiation.

In the in vivo evaluation, active osteoblasts were observed via H&E staining at the interface between the hydrogels and new bone, indicating ongoing healing and eventual complete repair of the bone defect [34]. The results of the in vivo study further supported the findings of cell viability, as rat calvarial defects implanted with the IPN-Li5 hydrogel scaffold exhibited higher bone volume fraction (BV/TV) at both four and eight weeks post-implantation compared to the other groups. MT staining is influenced by the molecular size of the anionic dye and tissue permeability. High molecular weight compounds can only penetrate porous and highly permeable tissues, whereas low molecular weight compounds can easily pass through dense structures with low tissue permeability. During the bone regeneration process, collagen becomes denser, more crosslinked, and arranged in a more regular manner, which affects collagen staining. Based on this principle, new early bone tissue is stained blue, while mature bone tissue is stained red [35,36,37]. The MT staining of the IPN-Li5 group exhibited a significant amount of red staining, indicating a greater quantity of mature new bone formation in that group. This observation was attributed to the deposition of collagen matrix, resulting in the reconstruction of new bone into regular lamellar bone [34]. Islands of new bone, not connected to the host bone, were also observed in the IPN-Li5 group after eight weeks of implantation. This phenomenon was attributed to the in situ mineralization of the implanted hydrogel, followed by remodeling by the host cells [38]. Furthermore, even after eight weeks of implantation, histological staining revealed the complete shape of the samples, indicating the structural integrity of the hydrogel scaffold. Combined with the degradation test results, the IPN hydrogel doped with lithium demonstrated its potential to serve as a long-term scaffold for bone regeneration.

## 4. Materials and Methods

### 4.1. Materials

Gelatin from porcine skin (Type A, 300 bloom, 50–100 kDa), methacrylic anhydride (MA), Irgacure 2959 (2-Hydroxy-4′-(2-hydroxyethoxy)-2-methylpropiophenone), and lithium chloride were purchased from Sigma-Aldrich (St. Louis, MO, USA). Sodium alginate with 300–400 cP, mannuronic acid content (M)/guluronic acid content (G) = 1.3 detected by ^1^H NMR, and MW = 4.2×10^5^ [39] was obtained from Wako (Osaka, Japanese). Calcium chloride was purchased from SHOWA Chemical Co., Ltd. (Tokyo, Japan).

### 4.2. Synthesis of GelMA

**Figure 10:**
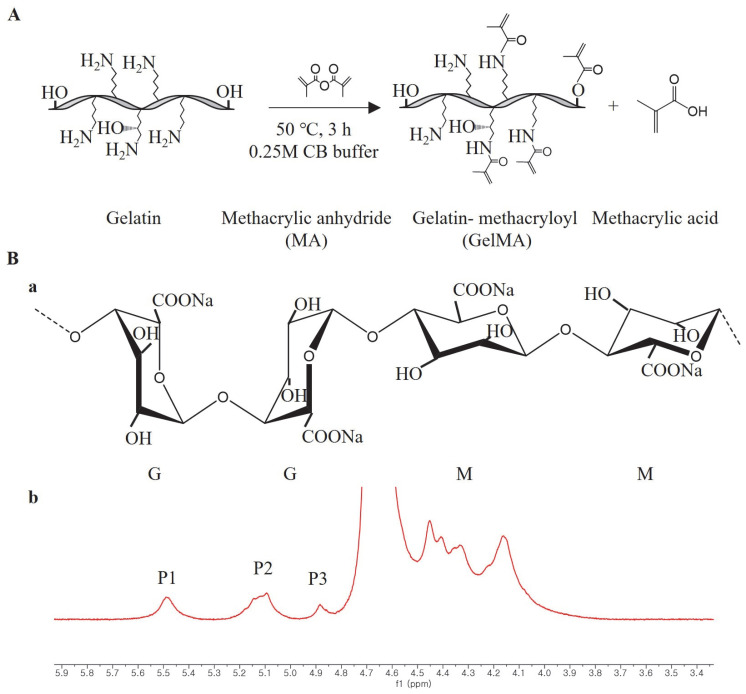
(**A**) Schematic of GelMA synthesis by gelatin and methacrylic anhydride (MA), (**B**) a, molecular structure of alginate (G and M represent β-L-guluronic acid and β-D-mannuronic acid, respectively); b, ^1^H NMR spectrum of sodium alginate in D_2_O at 80 °C, M/G ratio = [P2– P1+ P3]/P1 according to a previous study [41].

The facile one-pot synthesis method was used to synthesize GelMA in this study according to a previous study [28]. First, 0.25 M carbonate-bicarbonate (CB) buffer was prepared by dissolving 3.975 g sodium carbonate and 7.325 g sodium bicarbonate in 500 mL distilled water (DW), then 50 g gelatin was dissolved in 500 mL CB buffer, and the initial pH value was changed to 9 with 5 M HCl. After that, 5 mL MA was added to the mixture solution at 50 °C, and the pH value was changed to 7.4 with 5 M NaOH to stop the reaction after 3 h (Figure 10A). The potentially cytotoxic unreacted MA, salts, and byproducts in the mixture solution were removed through dialysis against DW, using a cutoff dialysis tube (12–14 kDa) at 37 °C for 7 days. Then, white foam-like GelMA was obtained after 7 days by lyophilization and stored at −20 °C before use.

The degree of substitution (DS) of the GelMA was quantified by ^1^H NMR spectroscopy according to a previous study [40]. ^1^H NMR spectra were collected using a 600 MHz FT-NMR spectrometer (JNM-ECZ600R, JEOL, Tokyo, Japan).

### 4.3. Synthesis of GelMA Alginate (SA) IPN and IPN-Li Hydrogel

The SA/GelMA IPN hydrogels were synthesized in three crosslinking steps, which were modified from our previous study (Figure 2A). First, 2.5% *w/v* SA and 20% *w/v* GelMA with 0.5% *w/v* photoinitiator were dissolved in DW with 5 or 10 mM LiCl at 40 °C, respectively. After that, SA and GelMA were mixed and transformed into a module immediately. The mixture was allowed to form a semi-IPN gel for 15 min under UV light exposure (WUV-L50, DAIHAN Scientific, Wonju, Republic of Korea) at 320–500 nm. Finally, the semi-IPN hydrogel samples were immersed in 2% *w/v* CaCl_2_ for 30 min to obtain the three crosslinked IPN hydrogels. The samples were divided into three groups according to the solvent and named IPN (DW), IPN-Li5 (5 mM LiCl), and IPN-Li10 (10 mM LiCl).

### 4.4. Physical and Mechanical Evaluation

The microstructure of the hydrogels was analyzed using variable pressure scanning electron microscopy (SU3900, Hitachi, Japan). The pore size and distribution were analyzed in SEM images using Image J software (National Institutes of Health, Bethesda, MD, USA) [42].

Fourier-transform infrared (FTIR) spectroscopy (Spectrum GX, Perkin Elmer, Waltham, MA, USA) was performed at the Center for University-wide Research Facilities (CURF) of Jeonbuk National University to determine the chemical bonding properties.

The compressive modulus of the hydrogel samples was determined using a compression testing system (Instron 5569, Instron, Norwood, MA, USA) with Bluehill software. In the course of the compression testing, the testing parameters were set as follows: 50 N cell loading, 0.5 mm/min crosshead speed, and 5 samples in each group. The compressive modulus was determined as the slope of the linear region corresponding to 5–15% strain in the stress–strain curve.

The swelling and degradation of the hydrogel samples were investigated using an immersion test. The samples were weighed after lyophilization (Wi). The lyophilized samples were then immersed in PBS and kept at 37 °C, and the immersion solution was replaced with fresh PBS weekly. Hydrogel samples were removed at specified time points (1, 7, 14, and 21 days) and weighed in the swollen state (Ws). The samples were then lyophilized and weighed again (Wd). The swelling and degradation ratios were calculated using the following equations:

Swelling ratio = Ws/Wi(1)

Mass loss = (Wi − Wd)/Wi × 100%(2)

The release of lithium ions was detected in an immersion experiment. Hydrogel samples were immersed in PBS and the immersion solution was collected and replaced with fresh PBS at different time points (0, 7, 14, and 21 days). All of the collected solutions were treated in the same way, with 0.6 mL HNO_3_ added to the collected immersion solution, mixed and heated at 80 °C for 10 h, and finally diluted tenfold with DW. The concentration of lithium ions released was measured using an inductively-coupled plasma mass spectrometer (ICAP RQ, Thermo Fisher Scientific, Waltham, MA, USA).

### 4.5. In Vitro

The cell culture media was prepared by adding 10% fetal bovine serum (FBS, Gibco Co. USA), 500 U/mL streptomycin (Gibco, Grand Island, NY, USA), and 500 unit/mL penicillin (Gibco, USA) to α-minimal essential media (MEM, Gibco, Carlsbad, CA, USA). MC3T3-E1 cells (2 × 10^4^ cells/mL, ATTC; American Type Culture Collection) were co-cultured with the hydrogels and the cell culture medium was changed every three days. Cells cultured without hydrogel were used as a control group. After 3 and 5 days, cell proliferation was detected using a CCK-8 kit (Enzo Life Sciences Inc., New York, NY, USA), and cell morphology was observed under a microscope after 5 days via crystal violet staining. Cell differentiation was detected using an ALP kit after 14 days of co-culture. As for the western blot experiment, co-cultured MC3T3-E1 cells were extracted with lysis buffer and the following antibodies were applied: p-GSK3β s9, β -catenin, Runx2, α-Tubulin (Cell Signaling Technology, Danvers, MA, USA), and osterix (Abcam, Cambridge, MA, USA). Protein bands were obtained after exposure to an imaging system (ImageQuant LAS 4000 mini, GE Healthcare, Chicago, IL, USA).

### 4.6. In Vivo

Male Sprague–Dawley rats, aged 8 weeks, were used as experimental subjects. All animal experiments complied with ARRIVE guidelines, were performed in accordance with the National Research Council’s Guide for the Care and Use of Laboratory Animals, and were approved by the Laboratory Animal Center of Jeonbuk National University, Jeonju-si, South Korea (approval number: NON2022-041-001). To investigate the bone repair ability of the hydrogel samples, two full-thickness defects (5 mm in diameter) were created in the parietal bones of rats. Prior to surgery, general anesthesia was induced by injecting an anesthetic agent consisting of Zoletil (0.06 mL/100 g, Zoletil 50, Virbac Laboratories, France) and xylazine hydrochloride (0.04 mL/100 g, Rompun; Bayer Korea Ltd., Republic of Korea). The hydrogel was sterilized with UV before implantation, and after implantation, the periosteum was sutured with bioabsorbable silk (5–0 glyconate monofilament, B. Braun, Rubí, Spain) to help fix the hydrogel, and then the skin was sutured with non-absorbable nylon silk (4/0 blue nylon, Ailee Co., Ltd., Busan, Republic of Korea). Amikacin (Samu Median Co., Ltd., Republic of Korea) was injected for three consecutive days to prevent infection.

After 4 or 8 weeks, the rats were sacrificed by euthanasia with an overdose of thiopental sodium (Choongwae Pharma Corporation, Seoul, Republic of Korea), and the collected tissues were analyzed by micro-CT (Skyscan 1076). The extent of reconstruction was assessed using Nrecon software. 3D and 2D analyses were performed using CT Analyzer software (SkyScan, Aartselaar, Belgium). The calvarial bone regeneration ability was evaluated by the bone volume density (BV/TV, %).

The specimens were treated with decalcifying agent (15% EDTA) and embedded in paraffin. The paraffin-embedded specimens were cut using a Leika system (HistoCore AUTOCUT, Hamburg, Germany) to a thickness of 6 μm. Hematoxylin and eosin (H&E) and Masson’s trichrome (MT) staining were performed using conventional methods and the specimens were examined under an optical microscope to determine new bone formation and bone maturation.

### 4.7. Statistics

One-way analysis of variance (ANOVA) with a 95% confidence interval was performed to evaluate statistical significance. All analyses were performed using GraphPad Prism 8.0.2 (GraphPad Software, La Jolla, CA, USA).

## 5. Conclusions

In this study, we successfully synthesized IPN hydrogels loaded with small molecules, capitalizing on the advantageous double-displacement reaction occurring between lithium ions and sodium alginate within the IPN hydrogel system. This reaction contributed to the notable enhancement of the physical properties of the IPN hydrogels. Moreover, through both in vivo and in vitro experimentation, we effectively demonstrated the ability of the IPN-Li5 hydrogel to promote bone regeneration. This favorable outcome can be attributed to the inherent stability of the IPN hydrogel, as well as the sustained release of lithium—a potent agonist of the Wnt/β-catenin signaling pathway. The findings from this study highlight the substantial potential of the IPN-Li hydrogel as a viable long-term clinical treatment option for bone defects. The combination of its structural stability and controlled release mechanism renders it a promising candidate for addressing such challenging medical conditions.

## Data Availability

Data are available on reasonable request.
